# Comparison on extreme pathways reveals nature of different biological processes

**DOI:** 10.1186/1752-0509-8-S1-S10

**Published:** 2014-01-24

**Authors:** Yanping Xi, Yue Zhao, Li Wang, Fei Wang

**Affiliations:** 1 Shanghai Key Laboratory of Intelligent Information Processing, Fudan University, Shanghai, 200433, China; 2 Department of Life Science, Fudan University, Shanghai, 200433, China

## Abstract

**Background:**

Constraint-based reconstruction and analysis (COBRA) is used for modeling genome-scale metabolic networks (MNs). In a COBRA model, extreme pathways (ExPas) are the edges of its conical solution space, which is formed by all viable steady-state flux distributions. ExPa analysis has been successfully applied to MNs to reveal their phenotypic capabilities and properties. Recently, the COBRA framework has been extended to transcriptional regulatory networks (TRNs) and transcriptional and translational networks (TTNs), so efforts are needed to determine whether ExPa analysis is also effective on these two types of networks.

**Results:**

In this paper, the ExPas resulting from the COBRA models of E.coli's MN, TRN and TTN were horizontally compared from 5 aspects: (1) Total number and the ratio of their amount to reaction amount; (2) Length distribution; (3) Reaction participation; (4) Correlated reaction sets (CoSets); (5) interconnectivity degree. Significant discrepancies in above properties were observed during the comparison, which reveals the biological natures of different biological processes. Besides, by demonstrating the application of ExPa analysis on E.coli, we provide a practical guidance of an improved approach to compute ExPas on COBRA models of TRNs.

**Conclusions:**

ExPas of E.coli's MN, TRN and TTN have different properties, which are strongly connected with various biological natures of biochemical networks, such as topological structure, specificity and redundancy. Our study shows that ExPas are biologically meaningful on the newborn models and suggests the effectiveness of ExPa analysis on them.

## Background

Many large-scale biological networks, including metabolic networks (MNs) [1], signaling networks [2], transcriptional regulatory networks [3] and transcriptional and translational networks [4] have been reconstructed along with the development of high-throughput technology in the past decades. These networks are then transformed into mathematical models for further analysis. Constraint-Based Reconstruction and Analysis (COBRA) is one of the most commonly used frameworks introduced to model and analyze steady-state biochemical networks [5]. In the past two decades, it has been successfully applied on MNs to study various phenotypes [6-9]. Recently, the same principles were also extended to other types of biochemical networks mentioned above [2-4,10].

All the possible phenotypes, i.e. the flux distributions of feasible steady states, of a constraint-based biochemical model form a high-dimensional cone. Network-based pathways such as Extreme Pathways (ExPas) [11] are defined to study this cone. ExPas are vectors of fluxes that lie on the edges of the cone [12]. They constitute the minimal and unique vector set which generates the space of all feasible steady states through non-negative linear combination. Since ExPas characterize the limits on the capabilities of a cell's metabolic system [13], ExPa analysis will reveal systemic properties of metabolism [14]. ExPa analysis as an approach to characterize the fundamental and time-invariant topological properties of a given network [15] has been successfully applied to MNs, such as those of human red blood cells [16], *Escherichia coli *[17-19], *Sacchoromryces cerevisiae *[20,21], *Helicobacter pylori *[22,23], *Haemophilus influenzae *[11,24] and *Methylobacterium extroguens *[25]. Besides, network models respectively describing a prototypic signaling system [10] and the JAK-STAT signaling system in the human B-Cell [2] have also been studied through ExPa analysis.

Recently, there emerged two COBRA models of biochemical systems with different types: *E.coli *transcriptional regulatory network (TRN) [3] and *E.coli *transcriptional and translational network (TTN) [4]. What should be clarified is whether ExPa analysis is still useful for new types of networks and whether ExPas of TRN or TTN show some properties different from those of MN. These questions are biologically significant because the answers determines whether we can rely on the existing analysis approaches to obtain novel and biologically meaningful findings in a brand new field. In this paper, we try to provide an anwser by comparing properties of ExPas among the *E.coli *TRN, MN and TTN. In the comparison, differences between biological processes were observed from multiple perspectives, including network structure, reaction participation, specificity and redundancy. The results indicate that ExPa analysis can be extended to biochemical systems of TRN and TTN, which helps researchers to further understand the corresponding biological systems. Besides an improved method was introduced to simplify the calculation and interpretation of ExPas on TRN models [3], which could also be useful.

## Results

Firstly, we calculated extreme pathways of the three biological networks mentioned above. Since the number of ExPas grows exponentially with a networks' complexity [15], the enumeration of ExPas on the highly complex ones such as *E.coli *MN and TTN is computationally intractable. Fortunately ExPa calculation will be much more manageable if a MN or a TTN is divided into smaller sub-networks. Therefore, we chose the sub-networks with relatively complete and independent functions as the representatives of their belonging biologic systems. For the *E.coli *MN, two sub-networks were chosen: (1) Amino acid, Carbohydrate and Lipid metabolism (sACL) and (2) Membrane and Murein metabolism (sMM). For *E.coli *TTN, the two sub-networks were: (1) transcription (sTC) and (2) translation (sTL).

Then ExPa analysis was performed on each network/subnetwork and the properties from different aspects were obtained, including the total number of ExPas, the number-based ratio of ExPa to reaction, ExPa length distribution, reaction participation distribution, correlated reaction sets (CoSets), and the inter-connectivity of ExPas. Finally, a horizontal comparison on the properties was made among the five networks/subnetworks.

Moreover, some incompleteness and incorrectness in the *E.coli *TRN model which were stumbled through ExPa analysis are also reported in this section. This findings illustrate that ExPa analysis is capable of directing model refinement.

### *E.coli *TRN model

The *E.coli *TRN model was published by Gianchandani et al. in 2009 [3]. It contains 147 environmental stimuli, 125 transcriptional factors and 503 downstream target genes which are represented in a matrix $R^{*}$[3]. The TRN model was improved to enhance the efficiency of ExPa calculation (Details are provided in *Materials and Methods*). The final TRN model contains 1009 components, 1106 internal regulatory reactions, and 1009 exchange reactions each corresponding to a component. All the extracellular metabolites were considered as inputs and all protein products were considered as outputs. There were 1599 ExPas, of which 9 were biologically infeasible because they employed conflicting input fluxes, and thus they were excluded from the ExPa set used in analysis.

In *E.coli *TRN, 16 reactions do not participate in any ExPa; namely they are never used to form a transcriptional state of the network. These unused reactions were categorized into two types as listed in Table 1 and Table 2 respectively.

**Table 1:** Unused reactions in the *E.coli *TRN (Type I - Regulatory rules missing).

Reactions	Reuglatory rules	Reaction type
NOT_BirA	--	internal
b0774_1	NOT(BirA)	internal
b0775_1	NOT(BirA)	internal
b0776_1	NOT(BirA)	internal
b0778_1	NOT(BirA)	internal
Ex_b0774	--	exchange
Ex_b0775	--	exchange
Ex_b0776	--	exchange
Ex_b0778	--	exchange

NOT_BirA represents the regulatory reaction that leads to the inhibition of gene transcription generating BirA. '-' in the second column represents the deficiency of corresponding regulatory rules in the model.

**Table 2:** Unused reactions in the *E.coli *TRN (Type II - Contradictory regulatory rules)

Reactions	Regulatory rules	Reaction type
b1814	( gly(e)>0) **AND **(leu-L(e)>0) leu-L(e)>0 (**NOT **o2(e)>0) **AND **(leu-L(e)>0) (gly(e)>0) **AND **(**NOT **leu-L(e)>0) **AND **(leu-L(e)>0) (leu-L(e)>0) **AND **(**NOT **leu-L(e)>0) (NOT o2(e)>0) **AND **(**NOT **leu-L(e)>0) **AND **(leu-L(e)>0)	Internal
b3942_1	(Growth>0) **AND **(h202(e)>0) **AND **(**NOT **Growth>0)	Internal
b4111_1	(**NOT**(Crp)) **AND **(Growth>0) **AND **(**NOT **Growth>0)	internal
Ex_b3942	--	Exchange
Ex_b4111	--	Exchange

*Crp *appears in the second column is a transcription factor and others are extracellular metabolites. The contradictory regulatory rules are labeled with shade in the table and these reactions never occur.

Reactions in Table 1 all relate to *NOT_BirA *(absence of protein *BirA*). However, no regulatory rule corresponds to the presence or absence of *BirA*, and therefore, the initial steps are unknown. As a result, the internal reactions using *NOT_BirA *(*b0774_1, b0775_1, b0776_1 *and *b0778_1*) and the corresponding exchange reactions (*Ex_b0774, Ex_b0775, Ex_b0776 *and *Ex_b0778*) will never be initiated. Furthermore, proteins *BirA *and the gene products of *b0774, b0775, b0776 *and *b0778 *do not participate in any other reaction except those in Table 1, so their invalidation will not affect other reactions in the network. In a word, these 9 reactions do not participate in any ExPa because their relevant reactions (either producing their substrates or consuming their products) are unavailable in the network. The unused reactions in Table 1 show the incompleteness of the *E.coli *TRN model and necessitate further refinement.

For the reactions in Table 2, the regulatory rule of *b1814 *can be divided by simple logical transformation into 6 rules, of which 3 contradict with each other (the shaded parts in Table 2). Since there are still 3 operational regulatory rules relating to the transcription of *b1814*, its corresponding exchange reaction can be initiated. Similarly, the regulatory rules of *b3942 *and *b4111 *are both contradictory and cannot be used in any ExPa. These reactions may imply some incorrect information in the model. Therefore, new biological knowledge is needed to improve *E.coli *TRN.

### *E.coli *MN and TTN model

The MN model of *E.coli *K-12 MG1655, iAF1260, was published by Feist et al, in 2007 [26]. It includes the activities of 1260 open reading frames (ORFs). It consists of 1688 metabolites and 2382 reactions. The *E.coli *TTN model was published by Thiele et al. in 2009 [4]. It consists of 11991 components and 13694 reactions which give rise to 423 functional gene products [4]. Given the critical inherent problem of combinatorial explosion during ExPa calculation, *E.coli *MN and TTN were divided into small sub-networks depending on the reactions' functions [11]. Sub-networks as representatives of important biological processes were chosen.

The *E.coli *MN was divided into 6 discrete sub-networks with different functions: one for exchange reactions which transfer metabolites in and out of the metabolic system and the others for internal reactions. Each reaction was assigned to one of the six sub-networks, whose details are listed in Table 3. Two sub-networks, Amino acid, Carbohydrate and Lipid metabolism (sACL) and Membrane and Murein metabolism (sMM), lie in the central part of *E.coli *MN and form the basis of other biological processes, and therefore they were chosen as the representatives of *E.coli *MN for ExPa analysis.

**Table 3:** Sub networks of the *E.coli *MN

# Sub network	Name	Containing subsystems	No. of reactions
1	Amino acid, Carbohydrat and Lipid metabolism (sACL)	Alanine and Aspartate Metabolism, Cysteine Metabolism, Folate Metabolism, Glutamate Metabolism, Glycine and Serine Metabolism, Histidine Metabolism, Methionine Metabolism, Threonine and Lysine Metabolism, Tyrosine Tryptophan and Phenylalanine Metabolism, Valine Leucine and Isoleucine Metabolism, Arginine and Proline Metabolism, Citric Acid Cycle, Pentose Phosphate Pathway, Pyruvate Metabolism, Glyoxylate Metabolism, Methylglyoxal Metabolism, Glycolysis and Gluconeogenesis, Oxidative Phosphorylation, Glycerophospholipid Metabolism, Anaplerotic Reactions	741
2	Nitrogen and Nucleotide metabolism	Cofactor and Prosthetic Group Biosynthesis, Nitrogen Metabolism, Nucleotide Salvage Pathway, Purine and Pyrimidine Biosynthesis, tRNA Charging	445
3	Membrane and Murein metabolism (sMM)	Cell Envelope Biosynthesis, Lipopolysaccharide Biosynthesis Recycling, Membrane Lipid Metabolism, Murein Biosynthesis, Murein Recycling	358
4	Transport	Transport Inner Membrane,Transport Outer Membrane,Transport Outer Membrane Porin	849
5	Others	Alternate Carbon Metabolism; Inorganic Ion Transport and Metabolism; Unassigned	408
6	Exchange	Exchange reactions	304

The *E.coli *TTN model comprises of 27 biological processes and the details are provided in [4]. Each process was treated as a discrete sub-network. The largest two sub-networks, *Transcription *and *Translation*, were chosen for further ExPa analysis.

### ExPa counting

The total numbers of ExPas and the number-based ratios of ExPa to reaction (P/R) are listed in Table 4. P/R depicts the proportionality of the numbers of ExPas and reactions in a network. Table 4 shows that the P/Rs of sACL (33.44) and sMM (32.40) are much higher than those of TRN (0.75), sTC (0.12) and sTL (0.25), which are a consequence of the linear structures of TRNs and TTNs [3,4]. In contrast, MNs are in more complex interconnection with a large number of alternative pathways, and thus their P/Rs are much higher. The redundancy of ExPas increases a metabolic system's flexibility and fitness to sudden environmental changes [23,27]. These results illustrate the fundamental differences in topological structure and redundancy among the three types of networks.

**Table 4:** Network characteristics and ExPa calculation results

Network name	No. of reaction	No. of ExPa	P/R ratio
TRN	2115	1590	0.75
ACL	741	24778	33.44
sMM	358	11600	32.40
sTC	1276	154	0.12
sTL	7386	1871	0.25

Abbreviations for subsystems: TRN, transcriptional regulation; sACL, amino acid, carbohydrate and lipid metabolism; sMM, membrane and murein metabolism; sTC, transcription; sTL, translation.

### ExPa length

**Figure 1:**
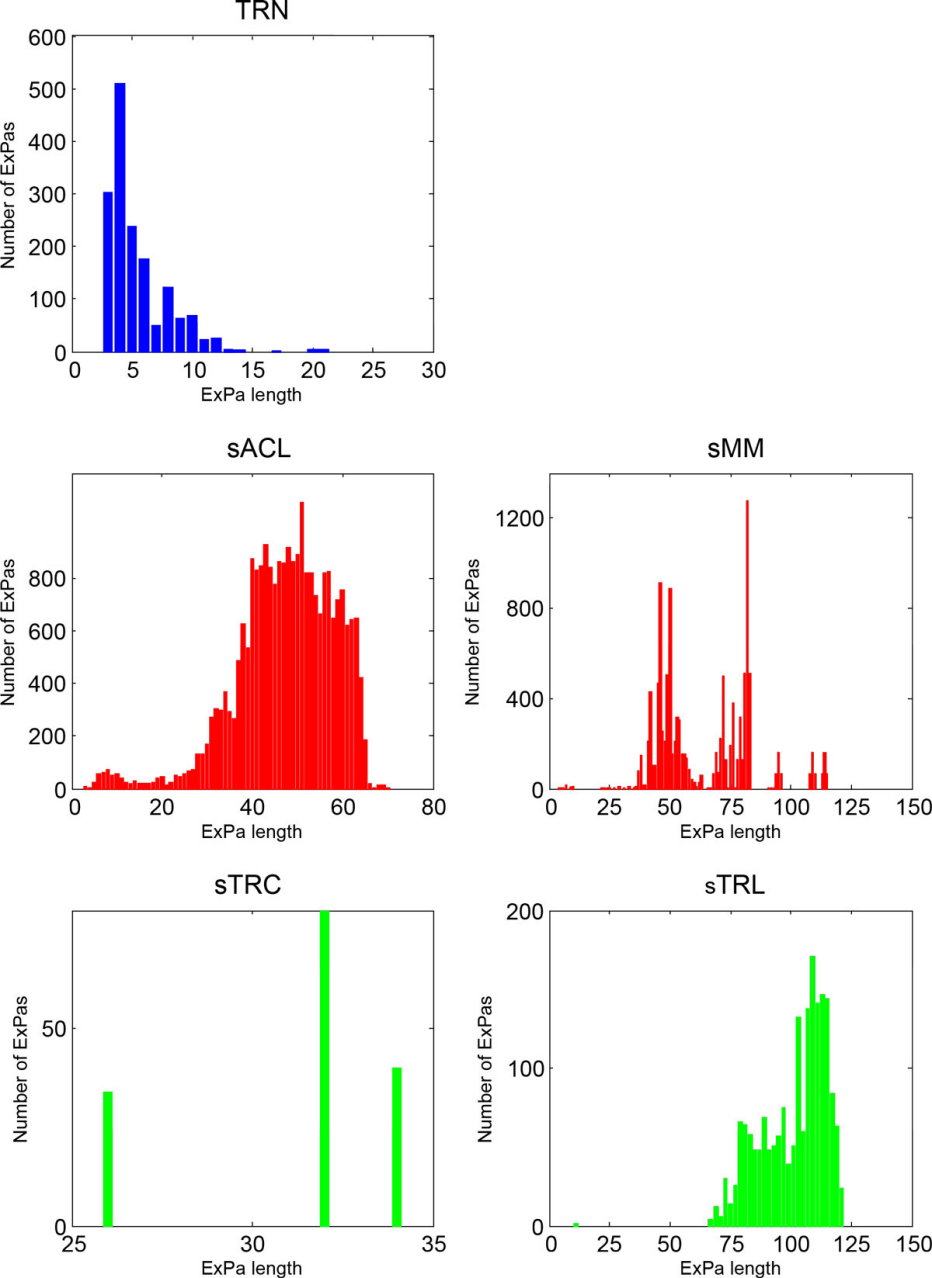
**ExPa length distributions in *E.coli *TRN, MN and TTN**. The x-axis represents the length of an ExPa. The y-axis represents the number of ExPas of the corresponding length.

The length of an ExPa equals to the number of reactions that participate in it [13]. Figure 1 shows the histograms of ExPa length distribution for each network/ sub-network above. The details are listed in Table 5.

**Table 5:** Summary of the statistical analysis of ExPa lengths

Network	No. of	ExPa length				L/R
			
	reactions	Avg	Max	Min	Most	Ratio(%)
TRN	2115	5	21	3	4	0.24
sACL	741	47	70	3	51	6.40
sMM	358	64	115	4	82	17.97
sTC	1276	31	34	26	32	2.43
sTL	7386	101	121	11	109	1.37

Abbreviations for subsystems: TRN, transcriptional regulation; sACL, amino acid, carbohydrate and lipid metabolism; sMM, membrane and murein metabolism; sTC, transcription; sTL, translation. 'Most' represents the ExPa length with the highest frequency. L/R ratio is the ratio of average ExPa length to reaction amount.

The length distributions of ExPas corresponding to those biological processes are very diverse. The longest ExPas consists 51, 82 32 and 109 reactions in sACL, sMM, sTC and STL, respectively, which is much longer than that in TRN (21). Reactions in *E.coli *TRN represent transcriptional regulatory rules rather than real biochemical reactions as in MN and TTN, and thus the ExPa length in TRN depicts the number of regulatory rules used for expressing certain genes. A regulatory rule describes how environmental stimuli affect transcriptional factors, which in turn affect downstream target genes. Therefore, the ExPa in TRN is reasonably shorter as the biological network has a relatively flat hierarchical structure [3]. Given the number of reactions, the ratio of average ExPa length to reaction number (L/R) was calculated for each biological network or subnetwork (Table 5). The L/Rs of the two representatives in MN are higher than those in TRN and their counterparts in TTN. Since ExPas convert substrates into products, ExPa length relates to how many reaction steps are needed to carry out the corresponding function. ExPa length can be characterized as the size and complexity of the corresponding flux distribution map [13]. The results indicate that the flux distribution map in MN is much more complex than those in TRN and TTN.

### Reaction participation

**Figure 2:**
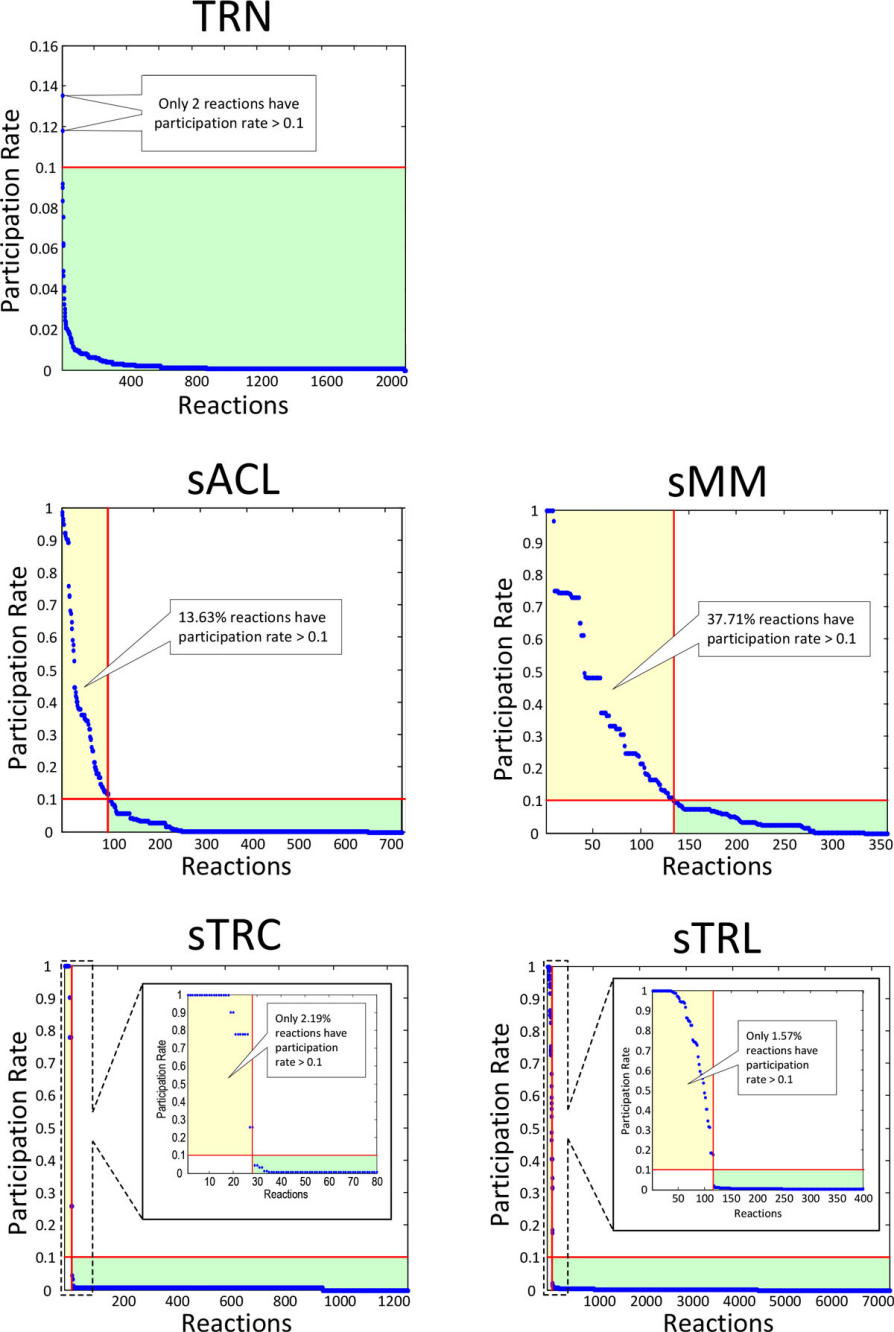
**Reaction participation distribution in *E.coli *TRN, MN and TTN**. Reactions are sorted in a descending order of ExPa participation rates. The x-axis represents the reaction rank. The y-axis represents the ExPa participation rate of reactions at corresponding rank.

The reaction participation rate (RPR) is defined as the percentage of ExPas in which a given reaction participates [13]. Figure 2 shows the distribution of RPRs for each biological network/sub-network. Most reactions participate in less than 10% of ExPas, especially in TRN, sTC and sTL, but a few active reactions participate in many ExPas. Although the high-RPR reactions are most exchange reactions, some of them are internal reactions which usually play a more important role in determining the phenotypic potentials of the five biological processes. Given this, RPR can be reasonably considered as a metric for evaluating the importance of a reaction to implement the corresponding biological function [13].

Here the top 10 internal reactions with the highest RPRs of each process are sorted in a descending order (Table 6). Several reactions of vital importance were found, and representatives were chosen for detailed study.

**Table 6:** The top 10 most frequently participated internal reactions

Part 1			
**The top 10 reactions with highest participation rate in the TRN**			

**Order**	**Reaction**		**Participation Rate (%)**

1	CRP_noGLC_1		9.18
2	Crp_1		8.99
3	NOT_PdhR_1		7.55
4	Fis_1		6.16
5	Lrp_1		4.09
6	Fnr_1		3.90
7	NOT_PurR_1		3.27
8	NOT_ArcA_1		3.02
9	NOT_Cra_1		2.83
10	NOT_Lrp_1		2.45

**The top 10 reactions with highest participation rate in the sACL**			

**Order**	**Abbr**.	**Reaction Name**	**Participation Rate (%)**

1	ASPTA	aspartate transaminase	91.42
2	ASAD	aspartate semialdehyde dehydrogenase	90.40
3	ASPK	aspartate kinase	90.40
4	HSDy	homoserine dehydrogenase NADPH	90.31
5	GHMT2r	glycine hydroxymethyltransferase reversible	75.82
6	GLYAT	glycine C acetyltransferase	68.22
7	HSK	homoserine kinase	67.27
8	THRS	threonine synthase	67.27
9	THRD	L threonine dehydrogenase	59.23
10	FUM	Fumarase	57.71

**The top 10 reactions with highest participation rate in the sMM**			

**Order**	**Abbr**.	**Reaction Name**	**Participation Rate (%)**

1	ACCOAC	acetyl CoA carboxylase	75.08
2	MCOATA	Malonyl CoA ACP transacylase	75.08
3	3HAD100	3 hydroxyacyl acyl carrier protein dehydratase n C100	74.48
4	3HAD40	3 hydroxyacyl acyl carrier protein dehydratase n C40	74.48
5	3HAD60	3 hydroxyacyl acyl carrier protein dehydratase n C60	74.48
6	3HAD80	3 hydroxyacyl acyl carrier protein dehydratase n C80	74.48
7	3OAR100	3 oxoacyl acyl carrier protein reductase n C100	74.48
8	3OAR40	3 oxoacyl acyl carrier protein reductase n C40	74.48
9	3OAR60	3 oxoacyl acyl carrier protein reductase n C60	74.48
10	3OAR80	3 oxoacyl acyl carrier protein reductase n C80	74.48

Part 2			

**The top 10 reactions with highest participation rate in the sTC**			

**Order**	**Abbr**.	**Reaction Name**	**Participation Rate (%)**

1	tscr_elo_TU-8389_ini	Formation complex for elongation of TU-8389'	0.65
2	tscr_elo_TU-8390_ini	Formation complex for elongation of TU-8390'	0.65
3	tscr_elo_TU-8397_ini_rho_dep	Formation complex for elongation of TU-8397 (RHO DEPENDENT TERMINATION)'	0.65
4	tscr_elo_TU-8407_ini_rho_dep	Formation complex for elongation of TU-8407 (RHO DEPENDENT TERMINATION)'	0.65
5	tscr_elo_TU0-1181_ini_stab	Formation complex for elongation of TU0-1181 (stable RNA)'	0.65
6	tscr_elo_TU0-1182_ini_stab	Formation complex for elongation of TU0-1182 (stable RNA)'	0.65
7	tscr_elo_TU0-1183_ini_stab	Formation complex for elongation of TU0-1183 (stable RNA)'	0.65
8	tscr_elo_TU0-1186_ini_stab	Formation complex for elongation of TU0-1186 (stable RNA)'	0.65
9	tscr_elo_TU0-1187_ini_stab	Formation complex for elongation of TU0-1187 (stable RNA)'	0.65
10	tscr_elo_TU0-1189_ini_stab	Formation complex for elongation of TU0-1189 (stable RNA)'	0.65

**The top 10 reactions with highest participation rate in the sTL**			

**Order**	**Abbr**.	**Reaction Name**	**Participation Rate (%)**

1	IF2_RECHARG	recharge of IF2 with GTP'	100
2	Rib_30_ini_FORM	formation of 30S translation initiation complex (30S subunit, IF1, IF2-GTP, IF3)'	100
3	Rib_70_DISS	70S ribosome dissociation'	100
4	EF-G_RECHARG	recharge of EF-G with GTP'	99.95
5	tl_elo_b3461_16_rib1	Translation elongation 1 b3461 16 ribosome(s)'	0.75
6	tl_elo_b3461_16_rib2	Translation elongation 2 b3461 16 ribosome(s)'	0.75
7	tl_elo_b3461_1_rib1	Translation elongation 1 b3461 1 ribosome(s)'	0.75
8	tl_elo_b3461_1_rib2	Translation elongation 2 b3461 1 ribosome(s)'	0.75
9	tl_elo_b3461_8_rib1	Translation elongation 1 b3461 8 ribosome(s)'	0.75
10	tl_elo_b3461_8_rib2	Translation elongation 2 b3461 8 ribosome(s)'	0.75

In TRN, the two most active reactions *CRP_noGLC_1 *and *Crp_1 *relate to the regulation rules of the transcription factor (TCF) C-reactive protein (CRP). Other high rank reactions *Fis_1, Lrp_1, Fnr_1*, and *NOT_ArcA_1 *relate to the regulation rules of the TCFs Fis, Lrp, Fnr and ArcA, respectively. In *E.coli*, the above TCFs belong to the seven global regulators that control most of the regulated genes [28]. The reaction *NOT_Cra_1 *is relevant to the regulation rules of the TCF Cra, a pleiotropic regulatory protein that controls carbon and energy fluxes in enteric bacteria [29,30]. The reaction *NOT_PdhR_1 *concerns the regulation rules of PdhR, a TCF that controls the respiratory electron transport system in *E.coli*. Its regulation target, the pyruvate dehydrogenase (PDH) multienzyme complex, plays a key role in the metabolic interconnection between glycolysis and the citric acid cycle [31].

In sACL, the most active reaction is *ASPTA*. It transfers oxoglutarate and aspartate to corresponding ketoacid, which are indispensable in glyoxylate cycle, an anabolic metabolic pathway occurring in *E. coli *[32]. The second one is *ASAD *which is the second step in the biosynthesis of amino acids in prokaryotes, fungi, and some higher plants. *ASAD *forms an early branch point in the metabolic pathway producing lysine, methionine, leucine and isoleucine from aspartate as well as diaminopimelate which plays an essential role in bacterial cell wall formation [33]. Deletion of gene *asd *(encoding *ASAD*) is lethal to the organism as demonstrated by experiments with *Legionella pneumophila, Salmonella typhimurium*, and *Streptococcus mutans*, which indicates that *ASAD *may also be an essential reaction in the metabolism of *E.coli *[34]. Another active reaction is *ASPK*, which is the commitment step in the pathway to the synthesis of lysine, methionine, threonine and isoleucine.

In sMM, the reaction *ACCOAC *is most active. It is a rate-determining step in the fatty acid synthetic pathway and may play a pivotal role in regulating fatty acid oxidation [35]. The second most active reaction *MCOATA *transfers Malonyl CoA to acyl-carrier proteins (ACPs). The product Malonyl ACP provides malonyl groups for biosynthesis of fatty acid and polyketide. On the other hand, Malonyl CoA, the substrate of *MCOATA*, is a highly-regulated molecule in fatty acid synthesis as it inhibits the rate-limiting step in beta-oxidation of fatty acids [36]. Flux change in *MCOATA *affects the consistency of Malonyl CoA and guarantees the biosynthesis of fatty acid.

In sTC, all the top reactions relate to the formation of the transcription elongation complex, an extremely complicated and highly regulated molecular machine that can sense signals coming from numerous regulatory protein factors, as well as those encoded in the DNA sequence. They are the basis of transcription elongation, because transcription can run smoothly and continuously only depending on their precise work.

In sTL, the reactions *IF2_RECHARG, Rib_30_ini_FORM *and *Rib_70_DISS *are used by all ExPas. *IF2_RECHARG *recharges the initiation factor 2 (IF2) with GTP and *Rib_30_ini_FORM *produces 30S translation initiation complex which consists of 30S subunit, IF1, IF2-GTP and IF3. In bacteria, the correct mRNA starting site and the reading frame are selected when, with the help of IF1, IF2 and IF3, the initiation codon is decoded in the peptidyl site of the 30S ribosomal subunit by the anticodon fMet-tRNAfMet. Furthermore, *Rib_30_ini_FORM *is also proved to be the intermediate step in the formation of 70S initiation complex (70SIC) which regulates translation initiation, the rate-limiting step in protein synthesis [37]. The other reaction *Rib_70_DISS *dissociates 70S ribosomes to 30S ribosomal subunit/IF1/IF3 complex (rib_30_IF1_IF3) and 50S ribosomal subunit (rib_50_inact). This is an essential step before a ribosome can participate in a new round of translation since the initiation complex for protein synthesis involves a 30S subunit. The dissociation of 70S ribosomes contributes to the efficiency and sustainability of protein synthesis [38].

Reportedly, RPRs help to find important reactions in MN [13]. Our results further indicate that RPR can also be extended to TRN and TTN to evaluate the relative importance of a given reaction.

### Correlated reaction set

A correlated reaction set (CoSet) comprises reactions that always participate in the same ExPa set in a given network [13]; namely if one reaction functions, the others in the same CoSet function simultaneously.

A CoSet can be transformed to a graph by treating each reaction as a node and adding an edge between two reactions that involve a common substance. In a certain CoSet, some member reactions are topologically connected while others are not. The correlationship of the second type of reactions often indicates a transcriptional coregulation by the corresponding genes [11] while that of the first type has relatively trivial biological meaning. Therefore, a CoSet is defined as a trivial set if all its member reactions are connected in topology. A trivial CoSet provides less novel information, and thus it is unworthy of deep study. In this paper, the adjacent ratio is used to represent the percentage of trivial CoSets.

CoSets were calculated for each biological network/sub-network about which several features, including the adjacent ratio, were stretched and shown in Table 7. The adjacent ratios of TRN, sTC and sTL are much higher than those of sACL and sMM, which indicates that almost all the CoSets obtained in the former three networks are due to the linear structure. For the metabolic netowrk, more CoSets consist of reactions which are not adjacent in topology. The results suggest that CoSet analysis may be more useful in study of MNs.

**Table 7:** Summary of CoSets

Network	No. of	No. of	Size of CoSet				Adjacent
				
	reactions	CoSet	Max	Min	Avg	Most	Ratio (%)
TRN	2115	481	8	2	2	2	98
sACL	741	157	11	2	2	2	78
sMM	358	49	25	2	4	3	47
sTC	1276	161	18	2	6	6	93
sTL	7386	1718	23	2	2	2	85

Adjacent ratio is defined as the percentage of such CoSets in which all the reactions are topologically connected, that is, if all the reactions in a CoSet form a graph, the graph only contains one connected component.

### Crosstalk analysis

Crosstalk analysis was first raised to illustrate the relationship between multiple inputs or outputs of a signaling pathway [39]. The whole ExPa set was compared pairwise to build the simplest form of crosstalk [2,10]. A pair of ExPas may have identical, overlapped or disjoint inputs (or outputs). There are 9 categories of crosstalk with their biological meanings described in [10]. Here, crosstalk analysis is applied to other biological processes to detect the relationships between fundamental functional states. Various forms of crosstalk in the five networks/sub-networks above were characterized. As several exchange reactions participate in most ExPas of sACL, MN, sTC and sTL, almost all of the ExPa pairs have overlapped inputs or outputs. A close look at the highly participating exchange reactions reveals that most of them relate to small molecules such as H_2_O, ATP and NADP commonly seen in various biochemical reactions. In order to further elucidate the difference in crosstalk between ExPa pairs, all the exchange reactions in the four sub-networks were sorted in a descending order depending on RPR and the top 20% ExPa pairs were neglected in the subsequent crosstalk analysis.

**Figure 3:**
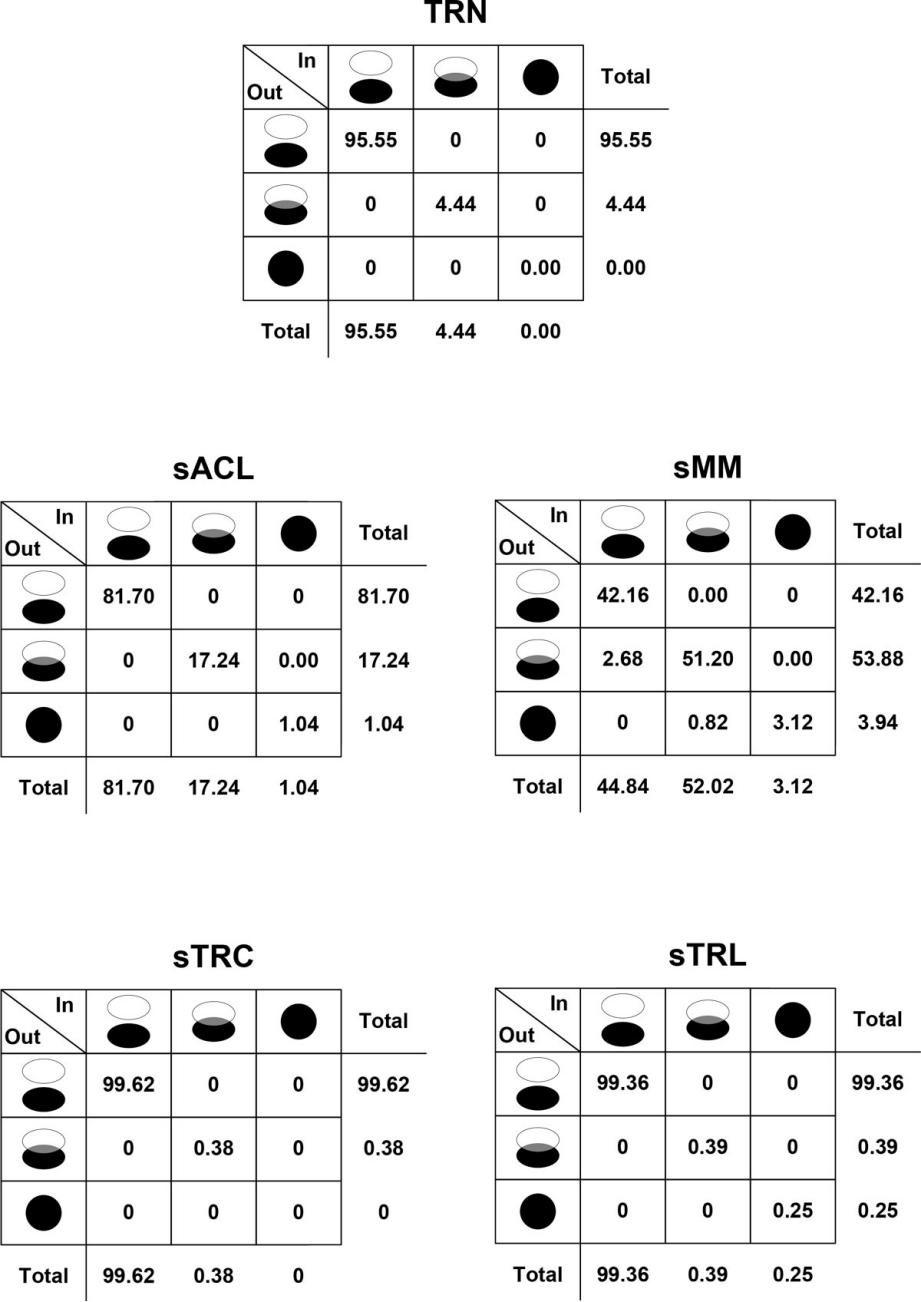
**Crosstalk analysis of *E.coli *TRN, MN and TTN**. Given a pair of ExPas, the relationship of their input sets falls into one of the following three cases: disjoint, partially overlapped and identical. And so does that of their output sets. Thus, all pairs of ExPas can be classified into 9 categories according to the relationship of input/output sets. Classification results of different networks are shown in this figure by 3x3 matrices in which the number in each cell represents percentages of ExPa pairs falling into this category.

As shown in Figure 3, more than 90% of the ExPa pairs have disjoint inputs and disjoint outputs in TRN, sTC and sTL in contrast to sACL and sMM. A higher disjoint input/disjoint output rate implies that each ExPa has more specific functions and cannot be replaced easily by others. This indicates that the biological processes in *E.coli *TTN and TRN are more deterministic than those in MN. Reportedly, a large number of genes are regulated by only a few independent regulatory rules in *E.coli *TRN [3], and the majority of the associated functions in *E.coli *TTN have only one coding gene in the genome [4]. These facts indicate that the specificity of TRN and TTN is much higher than MN. In order to function normally, cells have to respond accurately to the environmental signals with the help of precise transcriptional regulations and subsequently produce necessary gene products through accurate transcription and translation systems.

**Figure 4:**
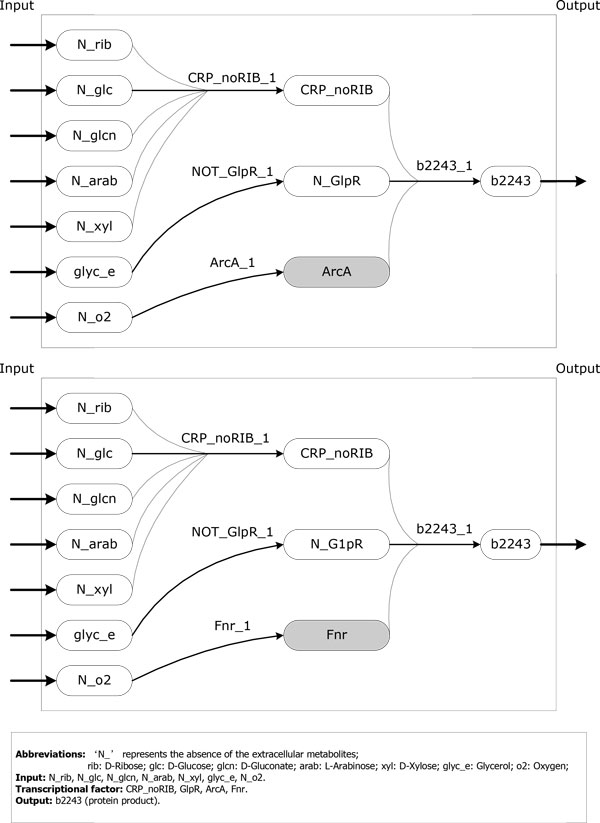
**Example ExPas with identical inputs and identical outputs**. The ExPa is represented by a DAG (Directed Ascyclic Graph), in which each node represents a component and each edge represents a reaction. The shaded part indicates the difference between two ExPas.

Except sTC, the other networks/sub-networks all have ExPa pairs with identical inputs and identical outputs. These ExPas are redundant pathways which fulfill completely identical function through systemically independent routes. ExPa redundancy was demonstrated in genome-scale MNs [23,24], as well as a prototypic signaling network [10] and the JAK-STAT signaling network [2]. The redundant ExPas in *E.coli *TRN can be attributed to the fact that the transcription of some genes can be stimulated by different transcriptional factors. For example, two redundant ExPas shown in Figure 4 stimulate the expression of gene *b2243 *in the same environment, but they employ the regulatory rules of '*CRP_noRIB AND Fnr AND NOT(GlpR)*' and '*CRP_noRIB AND ArcA AND NOT(GlpR)*', respectively. From Figure 3, the percentage of ExPa pairs with overlapped inputs and overlapped outputs in the biological processes of MN is much higher than those in TTN and TRN. These results indicate that *E.coli *MN is more flexible than TTN and TRN.

## Discussion

ExPa analysis were applied to two new models, the *E.coli *TRN and TTN. A horizontal comparison was performed for the five networks/sub-networks: TRN, sACL, sMM, sTC, sTL from five aspects: (1) Total number of ExPas and the P/R ratios; (2) ExPa length distribution and L/R ratios; (3) Reaction participation rates; (4) Correlated reaction sets and adjacent ratios; (5) Inter-connectivity of ExPas.

Reactions in TTN represent actual biochemical reactions like those in MN, and thus, ExPas in TTN characterize the steady-states of the corresponding biological systems. In contrast, columns in TRN represent the transcriptional regulatory rules and coefficents only reflect the qualitative information describing the presence or absence of the corresponding components rather than the quantitative information describing reaction stoichiometries as in TTN and MN. Therefore, an ExPa in TRN characterizes a specific transcriptional regulatory state, namely which transcriptional regulatory rules are activated and which genes are expressed in a specific environmental state.

ExPa analysis emphasizes the functional and systemic properties of biologcial process as ExPas are systemically independent functional units. The total number of ExPas and the P/R ratios characterize the flexibility of the networks/sub-networks. ExPa length corresponds to the reaction steps needed to form a steady state, therefore showing a close relation to network complexity. Crosstalk enables the analysis of pathway redundancy and network determinacy. Comparisons from these aspects indicate that MN is more flexible but less deterministic than TRN and TTN. Environmental cues affect transcriptional regulation, which controls the following transcription and translation processes. Then the resulting gene products (enzymes) enter the metabolic system to catalyze the corresponding reactions. It is necessary for a cell to respond accurately to the environment and produce the required enzymes. MN is more robust to environmental changes, which reflects the struggle of a cell to achieve an alternative steady-state to provide substance support for TRN and TTN and maintain life.

The distributions of reaction participation in the five networks/sub-networks are similar except that there are more reactions participating in more than 10% ExPas in sACL and sMM. Only a small percent of the reactions participate in a large number of ExPas, which indicates the phenotypic potentials of TRN, TTN and MN are affected greatly by a small number of important reactions. Evaluations on the representatives show that reactions with high participation rates often play an important role in certain biological processes. These reactions are the relatively weak part of the networks because a large number of ExPas will be destroyed when these reactions become invalid, which may cause the loss of various functions. These reactions may be used as drug targets and further direct the design of new drugs.

CoSets were identified via the calculation of reaction participation. Besides the expected topological connections, the topologically unconnected reactions in a CoSet may indicate the information of transcriptional coregulation in MN. However, most Cosets of TRN and TTN are trivial, and thus have few chances to be a clue giving novel information like in MN.

Last but not least, an improved approach was introduced to calculate the ExPas on TRN models. Compared to the existing method, the biggest advantage of ours is the high efficiency in calculating all the extreme pathways of a TRN, especially for the one which may work under huge amount of environmental conditions. For example, the *E.coli *TRN model which we studied in the paper has 776 components whose availability (i.e., presence or absence) constitute the environmental condition, including environmental stimuli, transcription factors or proteins. It is impossible to enumerate all the possible conditions due to "combination explosion" without mentioning the calculation of the ExPas under each condition. However, using the approach we proposed, it took only about 45 seconds to computing the whole ExPa set on a PC with four 3.2-GHz Intel(R) XEON processors and 16GB RAM (in fact, only one processor and 15MB RAM are used for the calculation). We believe that this approach could be helpful for readers who are also interested in the ExPas of TRNs.

## Conclusions

This study presents the first horizontal comparison among the E.coli TRN, MN and TTN through ExPa analysis. The results show that ExPa also has biological meanings in TRN and TTN. Different properties of ExPas reflect the biological nature of each biological process. Along with the the increase of reconstructed models on TRNs and TTNs as well as the development of new methods, ExPa analysis may reveal more biological properties and get larger space of application in the medical and biochemical fields.

## Methods

### COBRA framework and ExPa analysis

The COBRA framework stoichiometrically represents a biochemical network as a matrix  S, whose rows and columns correspond to components and reactions respectively. COBRA is capable of predicting and understanding the achievable cellular function, namely the phenotypic behavior of a biochemical network. With the hypothesis of steady state and certain constraints, all possible flux distributions lie in the null space of  S:

$$\text{S}v=0\text{, }v_{i}^{\text{min}}\leq v_{i}\leq v_{i}^{\text{max}}\text{, }i=1,\cdots \;,n$$

where ${\text{S}}_{m\times n}$ is the stoichiometric matrix of a biochemical network with  components and  reactions and  is a vector of the fluxes through each reaction in the system [40].

Given the reversibility of reactions, an internal reversible reaction can be divided into a forward and a backward sub-reactions, each taking a non-negative flux. The model's solution space is now a convex polyhedral cone in high-dimensional space [19,40], which can be demarcated by an ExPa set ${\text{p}}_{i}\left(i=1,\cdots \;,k\right)$[11,41]. All steady-states lie in the cone and each can be represented by a nonnegative linear combination of ExPas:

$$\text{v}=\sum \alpha_{i}{\text{p}}_{i}\;\;\;\text{ where }\;\;\;\alpha_{i}\geq 0$$

For a given network, the ExPa set has the following properties: (1) It is unique; (2) Each ExPa uses fewest reactions to form a function unit; (3) It is systemically independent which means an ExPa cannot be represented by a nonnegative linear combination of other ExPas [42,43].

### ExPa calculation on the MN and TTN

ExPas were calculated using an open source tool '*expa*' [44]. The *E.coli *MN and TTN models were divided into small sub-networks using the method proposed in [11].

### An improved approach to compute the ExPas of TRN models

A TRN is composed of a set of transcriptional regulatory rules which describe cells' transcriptional responses to environmental signals. A regulatory network matrix  was used by Gianchandani et al. to represent the components (environmental cues, metabolites, genes and proteins) and reactions (regulatory rules and exchange reactions of products) of a TRN [3]. It was further combined with an environmental matrix  E, which characterizes a particular environmental state, yielding a complete regulatory state matrix . Each column of  delineates the availability of a unique environmental cue, transcription factor, target gene or protein [3,45]. Different environmental states correspond to different s, thus forming different s.

For example, given a toy TRN with three regulatory rules:

A+B→Protein 1;C→Protein 2;D→Protein 2;

where A, B, C and D are four metabolites enacting as signalling stimuli.

**Figure 5:**
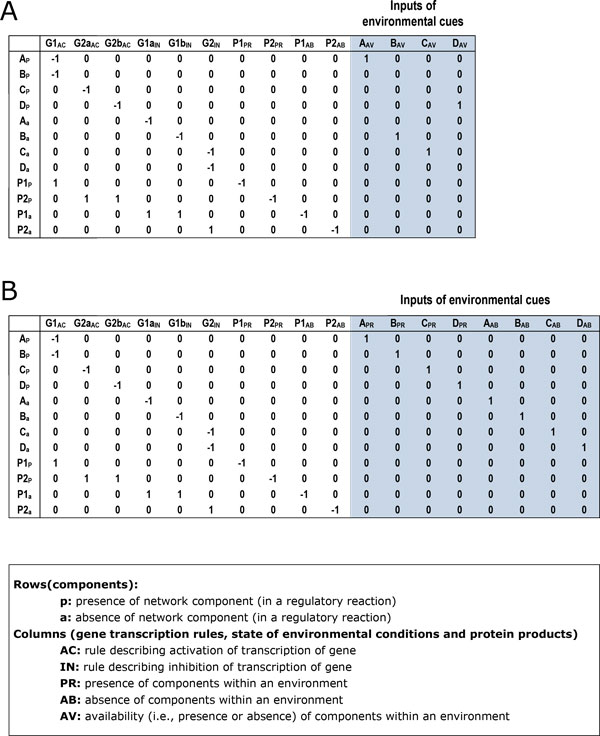
**Matrix formalism of the TRN model**. (A) The regulatory state matrix $R^{*}$ of the toy model in which the regulatory rules are: A+B→Protein 1; C→Protein 2; D→Protein 2. The environmental state of $R^{*}$ is that metabolites A and D are present while B and C are absent. (B) The corresponding ${\text{R}}_{\text{new}}^{*}$ of $R^{*}$.

The corresponding converses are:

A→Protein 1;B→Protein 1;C+D→Protein 2;

The matrix $R^{*}$ is illustrated in Figure 5A under the environmental condition that A and D are present while B and C are absent. The shaded columns represent the inputs of environmental cues. Any steady state of TRN under the given environmental cues lies in the space which satisfies $R^{*}\text{v}=0$ and $\forall i,v_{i}\geq 0$. The convex basis of the right null space of $R^{*}$ forms the ExPa set under the given environmental state.

In order to calculate all the ExPas of the TRN, all the environmental states, namely all possible s, need be enumerated. Then ExPas participating in each possible environmental state are generated and the unique ones are grouped to form the complete ExPa set. Since the number of possible environmental states grows exponentially with the number of extracellular metabolites, it is inefficient to enumerate all possible environmental states for a TRN with numerous envionmental cues [45]. Therefore, an improved method is introduced here to simplify the ExPa calculation on the COBRA model of TRN.

The gist of the method is to improve Gianchandani's method by employing two columns instead of one to delineate the presence and absence of a unique envionment cue respectively, by which a new environment matrix $E_{new}$ is constructed. The matrix $E_{new}$ covers all possible environmental states. Without loss of generality, we assume that the top  rows in  and  represents the present state of *n *environmental inputs $m_{e}\;\left(e=1,\cdots \;,n\right)$ one-to-one and the following  rows represents the absent state of them. The original regulatory state matrix is  and the new matrix is ${\text{R}}_{new}^{*}=\left[R|E_{new}\right]=\left[R_{1},R_{2}\cdots R_{k}|R_{k+1},R_{k+2},\cdots \;,R_{k+n}|R_{k+n+1},R_{k+n+2},\cdots \;,R_{k+2n}\right]$( is the number of columns in , and $E_{new}=[r_{k+1},r_{k+2},\cdots \;,r_{k+n}|r_{k+n+1},r_{k+n+2},\cdots \;,r_{k+2n}]$). For an input $m_{\text{e}}$, column  represents its presence and column  represents its absence under the environmental condition, where $R_{k+e}\left(e\right)$ and $R_{k+n+e}\left(n+e\right)$ equal to 1 and the other elements are all zeros. For example, the ${\text{R}}_{new}^{*}$ matrix of the above toy model is illustrated in Figure 5B. The shaded columns constitute $E_{new}$. Obviously, the space and time complexity for constructing $E_{new}$ is O(n), where  is the number of components of a TTN model. The convex basis of the right null space of  comprises the ExPa set of the TRN which could then be enumerated by the tool '*expa*' [44].

**Figure 6:**
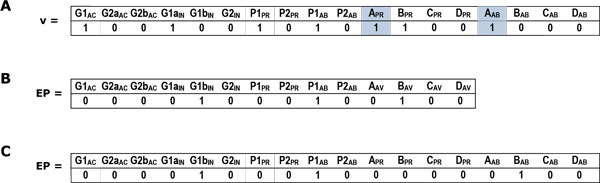
**Example ExPas of TRN**. (A) An example of infeasible regulatory state in the null space of ${\text{R}}_{\text{new}}^{*}$ in Figure 5B. The shaded parts indicate the contradictory inputs of state . (B) An example ExPa resulting from the matrix $R^{*}$ in Figure 5A. (C) The same ExPa as that in (B) resulting from ${\text{R}}_{\text{new}}^{*}$ of Figure 5B.

Notably, some infeasible steady states employing contradictory inputs may be involved in the right null space of ${\text{R}}_{new}^{*}$. For example, Figure 6A shows an infeasible steady sate of the TRN described in Figure 5B. The two shaded elements of  both equal to 1. This means metabolite A is both present and abscent in the environment, which is obviously impossible. If an ExPa proves to be an infeasible steady state, it should be removed from the ExPa set.

Figures 6B and 6C show two ExPas resulting from the matrixs in Figures 5A and 5B respectively. The two vectors represent the same steady state of the TRN in which gene G1 is inhibited because of lack of metabolite B. In Figure 6B, the exact meaning of "" in element $B_{AV}$ cannot be decided directly from ExPa without referring to the shaded part of matrix in Figure 5A. However, in Figure 6C, "" in column  clearly means the absence of metabolite . Namely, the interpretation of an ExPa resulting from the improved method is independent from the environmental matrix, which makes an ExPa easier to understand.

### Validation of the approach of ExPa calculation on TRNs

Given  environmental cues, there are $2^{n}$ possible environmental states, each corresponding to a matrix $E_{i}$ and the corresponding ${\text{R}}_{i}^{*}$ (${\text{R}}_{i}^{*}=[\text{R}|E_{i}]$, $i=1,\cdots \;,2^{n}$). The ExPa set obtained from ${\text{R}}_{i}^{*}$ is denoted as ${\text{P}}_{i}$ and the feasible ExPa set calculated from ${\text{R}}_{new}^{*}$ is denoted as ${\text{P}}_{new}$. Since the meaning of the environmental part of ${\text{P}}_{i}$ is dependent on the environmental states, ExPas of different environmental states should be normalized to eliminate the dependence before being grouped up. We normalized a ExPa ${\text{p}}_{i}^{j}$ in the set ${\text{P}}_{i}$ by expanding its dimension of the input part from  () to 2n (${\hat{\text{p}}}_{i}^{j}$). Details of the normalization are described in *Algorithm 1*.

**Data: **${\text{p}}_{i}^{j}$,$A^{i}$,${\bar{A}}^{i}$.

// ${\text{p}}_{i}^{j}$ represents the th ExPa in the th environment, where ;

// ${\text{p}}_{i}^{j}=\left[v_{1},v_{2},\cdots \;,v_{k}^{}|v_{k+1},v_{k+2},\cdots \;,v_{k+n}\right]$;

// $A^{i}$ is a set which consists of all the absent inputs;

// ${\bar{A}}^{i}$ is a set which consists of all the present inputs;

**Result: **${\hat{\text{p}}}_{i}^{j}$. // ${\hat{\text{p}}}_{i}^{j}=\left[v_{1},v_{2},\cdots \;,v_{k}^{}|v_{k+1},v_{k+2},\cdots \;,v_{k+n}^{},v_{k+n+1},v_{k+n+2},\cdots \;,v_{k+2n}\right]$;

**For **q=1**to ****do **${\hat{\text{p}}}_{i}^{j}\left(q\right)={\text{p}}_{i}^{j}\left(q\right)$; **End for**

**For **q=1**to **n**do**

    **If **${\text{p}}_{i}^{j}\left(k+q\right)=0$**do**

        ${\hat{\text{p}}}_{i}^{j}\left(k+q\right)=0;\;{\hat{\text{p}}}_{i}^{j}\left(k+n+q\right)=0;$

    **Else if **${\text{p}}_{i}^{j}\left(k+q\right)=1$**do**

        **If **$q\in A^{i}$**do**

            ${\hat{\text{p}}}_{i}^{j}\left(k+q\right)=0;\;{\hat{\text{p}}}_{i}^{j}\left(k+n+q\right)=1;$

        **Else if **$q\in {\bar{A}}^{i}$

            ${\hat{\text{p}}}_{i}^{j}\left(k+q\right)=1;\;{\hat{\text{p}}}_{i}^{j}\left(k+n+q\right)=0;$

        **End if**

    **End if**

End for

**Algorithm 1**: Procedure of normalizing ${\text{p}}_{i}^{j}$ to ${\hat{\text{p}}}_{i}^{j}$ by dimension expanding.

In a normalized ExPa $\hat{\text{p}}=\left[v_{1},v_{2},\cdots \;,v_{k+2n}\right]$, "" on $v_{k+e}\;\left(e=1,\cdots \;,n\right)$ indicates that $m_{e}$ is present on the ExPa while "" on  indicates that $m_{e}$ is absent, and  indicates that  does not affect the transcriptional states characterized by this Expa. The normalized ExPa set of ${\text{P}}_{i}$ is denoted as  and the union of ${\hat{\text{P}}}_{i}$$\left(i=1\ldots 2^{n}\right)$ is denoted as $\hat{\text{P}}$. As explained above, the ExPas in set ${\text{P}}_{new}$ are already in the normalized form, hence no normalization are needed.

Here we prove that ${\hat{P}}_{new}$ equals to $\hat{\text{P}}$:

**Statement 1**: each ExPa in $\hat{\text{P}}$ can be obtained from ${\text{R}}_{new}^{*}$.

**Proof: **given  extracellular metabolites $m_{p}\left(p=1,\cdots \;,n\right)$, each ${\text{R}}_{i}^{*}$ can be transformed to ${\hat{\text{R}}}_{i}^{*}$ as follows (**Algorithm 2**):

**Data: **${\text{R}}_{i}^{*}$,$A^{i}$,${\bar{A}}^{i}$.

// ${\text{R}}_{i}^{*}$ represents TRN in the th environment, where $i=1,\cdots \;,2^{n}$;

// ${\text{R}}_{i}^{*}=\left[R_{1},R_{2},\cdots \;,R_{k}|R_{k+1},R_{k+2},\cdots \;,R_{k+n}\right]$;

// $A^{i}$ is a set which consists of all the absent inputs;

// ${\bar{A}}^{i}$ is a set which consists of all the present inputs;

**Result: **${\hat{\text{R}}}_{i}^{*}$.

// ${\hat{\text{R}}}_{i}^{*}=\left[R_{1},R_{2},\cdots \;,R_{k}|R_{k+1},R_{k+2},\cdots \;,R_{k+n},R_{k+n+1},R_{k+n+2},\cdots \;,R_{k+2n}\right]$;

**For **q=1**to **2n**do **$R_{k+q}=0$; **End for**

**For **q=1**to **n**do**

    **If **$q\in {\text{A}}^{i}$**do**

        $R_{k+n+q}\left(n+q\right)=1$;

    **Else if **$q\in {\bar{A}}^{i}$

        $R_{k+q}\left(q\right)=1$;

    **End if**

End for

**Algorithm 2: **Procedure of transforming ${\text{R}}_{i}^{*}$ to ${\hat{\text{R}}}_{i}^{*}$.

For ${\hat{\text{R}}}_{i}^{*}=\left[R_{1},R_{2},\cdots \;,R_{k}|R_{k+1}\text{,}R_{k+2},\cdots \;,R_{k+n},R_{k+n+1},R_{k+n+2},\cdots \;,R_{k+2n}\right]$ ($i=1,\cdots \;,2^{n}$) resulted from Algorithm 2, if ∃j∈{k+1,⋯,k+2n} such that $R_{j}=0$, then a constraint $v_{j}=0$ is added. Then the resulting network is a sub-network of that represented by ${\text{R}}_{new}^{*}$. As proven in [46],  and $G^{\prime }$ are two MNs whose reactions are all irreversible and whose ExPa sets are  EP and $\text{E}{\text{P}}^{\prime }$, respectively. If $\text{E}{\text{P}}^{\prime }$ is a sub-network of  EP, then $\text{E}{\text{P}}^{\prime }\subseteq \text{EP}$. Therefore ${\hat{\text{P}}}_{i}\subseteq {\hat{\text{P}}}_{new}$, because $\text{P}=\overset{n}{\underset{i=1}{⋃}}{\hat{\text{P}}}_{i}$, $\hat{\text{P}}\subseteq {\hat{\text{P}}}_{new}$.

**Statement 2: **each feasible ExPa in ${\hat{\text{P}}}_{new}$ can be obtained by some ${\hat{\text{R}}}_{i}^{*}$.

**Proof: **Since any environmental cue is impossible to be both present and absent in a specific environment, $v_{k+e}\times v_{k+n+e}=0$ (e=1,⋯,n) is true for each ExPa in $P_{new}$. For any ExPa $\text{p}\in {\hat{P}}_{new}$, let $T={\text{R}}_{new}^{*}$. For any ,  is modified as follows: (1) If $v_{k+e}=0$ and $v_{k+n+e}\neq 0$, $t_{k+e}=0$; (2) If $v_{k+n+e}=0$ and $v_{k+e}\neq 0$, $t_{k+n+e}=0$; (3) If $v_{k+e}=0$ and $v_{k+n+e}=0$, $t_{k+e}=0$, where $t_{i}$ is the th column of . As can be shown easily,  is an ExPa of the right null space of . According to *Algorithm 2*, a legal  contains one zero column and one non-zero column corresponding to the two input reactions of a certain input component respectively. Therefore,  is a legal , and each ExPa in  can be obtained by some ${\hat{\text{R}}}_{i}^{*}$, or in other words, ${\hat{\text{P}}}_{new}\subseteq \hat{\text{P}}$.

From statements (1) and (2), we conclude that ${\hat{\text{P}}}_{new}=\hat{\text{P}}$, and thus all possible ExPas of a TRN can be obtained using our new representation.

### Classification of ExPas

ExPas fall into three classes, in which class III stands for internal reaction cycles with no exchange flux [12]. Class III ExPas were proven to be thermodynamically infeasible [47] and thus were not considered in our analysis.

## List of abbreviations used

COBRA: Constraint-based Reconstruction and Analysis; MN: Metabolic Network: ExPa: Extreme Pathway; TRN: Transcriptinal Regulatory Network; TTN: Transcriptional and Translational Network; sACL: The subnetwork of Amino acid, arbohydrate and Lipid metabolism; sMM: The subnetowrk of Membrane and Murein metabolism; sTC: The subnetwork of Transcription in the TTN; sTL: The subnetwork of Translation in the TTN; ORF: Open Reading Frame; P/R: the Number-based Ratios of ExPa to Reaction; L/R: the Ratio of Average ExPa Length to Reaction Number; RPR: the Reaction Participation Rate; TCF: Transcription Factor; CRP: C-reactive Protein; PDH: Pyruvate Dehydrogenase; ACP: Acyl-carrier Protein; IF: Initiation Factor; 70SIC: 70S Initiation Complex; rib_30_IF1_IF3: 30S Ribosomal Subunit/IF1/IF3 Complex; rib_50_inact: 50S Ribosomal Subunit; CoSet: Correlated Reaction Set.

## Competing interests

The authors declare that they have no competing interests.

## Authors' contributions

YX conceived and designed the study, participated in drafting and revising the manuscript. YZ carried out the analysis and drafted the manuscript. LW interpreted the results biologically. FW supervised the study, participated in its design and to revise the manuscript. All authors read and approved the final manuscript.

## Declarations

Publication of this article was funded by the corresponding author.

This article has been published as part of *BMC Systems Biology *Volume 8 Supplement 1, 2014: Selected articles from the Twelfth Asia Pacific Bioinformatics Conference (APBC 2014): Systems Biology. The full contents of the supplement are available online at http://www.biomedcentral.com/bmcsystbiol/supplements/8/S1.
